# Effect of passive motion on articular cartilage in rat osteoarthritis

**DOI:** 10.3892/etm.2014.1746

**Published:** 2014-05-29

**Authors:** JIE QIAN, JUN LIANG, YUBIN WANG, HUIFANG WANG

**Affiliations:** 1 Institution of Life Science and Technology, Tongji University, Shanghai 200092, P.R. China; 2 Department of Sport Medicine, Shanghai East Hospital, Tongji University School of Medicine, Shanghai 200092, P.R. China; 3 Department of Rehabilitation Medicine, Shanghai East Hospital, Tongji University School of Medicine, Shanghai 200092, P.R. China

**Keywords:** osteoarthritis, exercise, repair, histomorphology

## Abstract

The aim of the present study was to investigate the effect of moderate passive motion on articular cartilage in osteoarthritis (OA) caused by knee fracture. Sprague-Dawley rats (age, 8 weeks) with knee fractures were used to construct rat knee early- and middle-stage OA models. The stages were fixed for three and six weeks, with 20 rats analyzed at each stage. The experimental groups were exercised daily for 15 m/min with a specified duration. Following the completion of exercise, the effects of proper passive motion on cartilage thickness, the Mankin rating, cartilage collagen matrix, proteoglycan content and the morphological structure of the cartilage in the rat OA models were measured at the various degenerative stages caused by knee fracture. The proteoglycan content of the cartilage matrix, type II collagen fibers and the number of cartilage cells undergoing apoptosis were semiquantified. For early- and middle-stage OA, the cartilage layers in the three- or six-week experimental groups were significantly thicker and the levels of proteoglycans and type II collagen fibers in the weight-bearing area of the cartilage were significantly higher when compared with the control groups (P<0.05). In addition, the Mankin ratings were lower and ligament tension was increased when compared with the control group (P<0.05). In the early-stage OA group, significantly decreased apoptotic rates (P<0.05) were observed in the three- and six-week experimental groups, however, no significant decrease was observed in the middle-stage OA group. In the early-stage OA rats, the thickness of the cartilage layer, as well as the levels of proteoglycans and type II collagen fibers, in the six-week experimental group, were significantly higher compared with the control and three-week subgroups, and a decreased apoptotic rate was observed (P<0.05). In the six-week experimental middle-stage OA group, significant differences were observed in the content of proteoglycans and type II collagen fibers when compared with the control group, but not when compared with the three-week experimental group. Therefore, proper passive motion can repair and improve the metabolism of chondrocytes and delay the degenerative progress of articular cartilage in OA caused by knee fracture. However, for middle-stage OA, passive motion exhibits no significant repairing effect on the articular cartilage. This therapy increases the levels of proteoglycans and collagen fibers by reducing their decomposition, thereby improving the strength of the articular ligament and the stability of articulation.

## Introduction

Osteoarthritis (OA) is a type of chronic progressive osteoarthropathy that is common in middle-aged individuals. The prevalence of OA is increasing annually and has become the leading cause of joint pain and disability in the elderly. OA causes an enormous economic burden to patients, the family of patients and to society. Thus, an effective prevention of OA has become a major public health issue. A number of methods, including systemic treatment, medication and surgery, have been adopted over the past years, however, the clinical efficacy requires improvement (1).

In recent years, motion therapy has been applied extensively for OA treatment. Clinical and animal experiments have shown that regular motion therapy can relieve the symptoms of knee OA and improve knee function (2–4). However, research into motion therapy for the treatment of OA is limited, and there are a number of limitations associated, including whether exercise is effective in all types of OA or only to a particular type of OA. In addition, the effect of strength, frequency, opportunity and mechanism of motion therapy on OA treatment is unknown.

Running is a common exercise. In the present study, an OA degeneration model was constructed using knee fracture. A PT98 rat running device was used to simulate running exercise for early- and middle-stage OA. Staining techniques, namely, hematoxylin and eosin (HE), toluidine blue and immunohistochemistry, as well as transmission electron microscopy (TEM), were used to observe the effect of proper passive motion on cartilage thickness, the cartilage collagen matrix, protein proteoglycan content and the morphological structure of the cartilage in the OA rat model. The effect of proper passive motion on OA degenerative cartilage and other aspects were investigated, as well as the possible mechanism.

## Materials and methods

### Experimental animals

The study was approved by the Ethics Committee of Tongji University (Shanghai, China) and the study followed the animal care guidelines of Tongji University. In total, 40 Sprague-Dawley male rats (age, 8 weeks; weight, 200–220 g) that were specific-pathogen free, were provided by the Experimental Animal Center of Tongji University. Knee fracture (5) was used to construct early- and middle-stage rat articular OA degeneration models, which were fixed for three and six weeks, with 20 rats assigned for each group. The animals were divided randomly into exercise and control groups for each stage, from which each group was divided into three- and six-week exercise subgroups. In the exercise group, a PT98 electric animal treadmill was used at 15 m/min for 1 h/day. By contrast, rats in the control group were free following removal of the plaster. During the experimental process, the animals were housed separately and were provided with natural light and free access to a diet. Feeding conditions were maintained at 20–23°C, with a relative humidity of 50–55%.

### Sampling and handling for light microscopy

All the rats in the experimental groups were sacrificed at a set time. Cartilage tissues in the internal epicondyle of the femur were extracted and subjected to fixation, decalcification and progressive dehydration. The samples were sliced following embedding for HE, toluidine blue and immunohistochemical staining detection.

### Sampling and handling for TEM

Cartilage specimens were extracted by obtaining a 1×1×1 mm tissue section from the weight-bearing area of the condyle from the femur. The specimen was sliced into ultra-thin sections and was subjected to fixation, decalcification, gradient dehydration and embedment. Next, the specimen was observed with TEM, following double staining with uranygl acetate and 70% lead nitrate.

### HE staining

Surfaces of the articular cartilage, chondrocytes, cartilage matrix and the tidemark were observed with light microscopy. Two independent observers prepared slice ratings according to the Mankin rating (6), which were averaged to derive Mankin’s scores. Images were observed with light microscopy (magnification, ×10 and ×40) and were stored into an Image-Pro Plus 6.0 image analysis system (Media Cybernetics, Inc., Rockville, MD, USA) to determine the thickness of the cartilage layer (distance between the cartilage surface and the junction of the calcified cartilage and bone). In total, five specimens were obtained for each sample, five visual fields were collected for each specimen and the average value was computed.

### Toluidine blue staining, immunohistochemistry and terminal deoxynucleotidyl transferase dUTP nick end labeling (TUNEL) apoptosis assay

A type II collagen polyclonal antibody SP immunohistochemical staining kit and a TUNEL *in situ* apoptosis detection kit (Wuhan Boster Biological Technology, Co., Ltd., Wuhan, China) were used according to the manufacturer’s instructions.

An Image-Pro Plus 6.0 image analysis system was adopted and five visual fields were selected for each specimen. The average absorbance value of the positive staining in the unit area was measured to express the proteoglycan content in the cell matrix by semiquantitative measurements conducted with light microscopy (magnification, ×10 and ×40).

### TEM observations

TEM (JEM-1230; JEOL, Tokyo, Japan) was used to observe the cartilage surface, fiber and ultrastructure of the cells; images were captured.

### Biomechanical test

Knee articulation was applied to prepare a combination specimen of the femur and tibia (retention lengths of the femur and tibia were 3–4 cm from the knee articulation, soft tissues were removed and only the medial accessory ligament was retained). A CSS-44010 type electronic universal testing machine (Changchun Institute of Mechanical Science, Co., Ltd, Changchun, China) was used to stretch the specimen to cause rupture, from which the maximum load was obtained and was measured in Newtons.

### Statistical analysis

Statistical analysis was performed using SPSS 13.0 software (SPSS, Inc., Chicago, IL, USA). Data are expressed as the mean ± standard deviation. Comparisons between two groups were performed using the t-test, where P<0.05 was considered to indicate a statistically significant difference.

## Results

### Thickness of the articular cartilage and the Mankin rating

Thicknesses of the articular cartilage and the Mankin ratings of all the groups following passive motion are shown in Table I.

Results for the early-stage OA rats demonstrated that the cartilage thickness of the three-week group increased significantly when compared with the control group (P<0.05). In addition, the cartilage thickness of the six-week group increased significantly compared with the control and three-week groups (P<0.05). These results indicated that proper passive motion significantly increased the thickness of the articular cartilage in rats with early-stage OA that had been caused by knee fracture, and the effect was improved with prolonged exercise duration.

Mankin ratings of the two experimental early-stage OA groups were significantly lower compared with the respective control groups (P<0.05). However, no significant difference was observed between the two experimental groups. These results indicated that proper passive motion repaired and improved the structure and function of chondrocytes in rats with early-stage OA caused by knee fracture, as well as delayed the degeneration of articular cartilage.

In the middle-stage OA group, the cartilage samples in the three- and six-week groups were thicker compared with the control groups (P<0.05), indicating that proper passive motion increased the thickness of the cartilage in rats with middle-stage OA caused by knee fracture.

Mankin ratings of the two exercise middle-stage OA groups did not increase or decrease compared with the respective control groups (P>0.05). However, statistically significant differences were observed between the six- and three-week control groups, and between the six- and three-week experimental groups (P<0.05). These results indicated that proper passive motion improved the thickness of articular cartilage in rats with middle-stage OA caused by knee fracture. However, the repairing effect and improvements to the structure and function of the chondrocytes were not significant.

### Levels of proteoglycans and type II collagen fibers

Absorbance values from the toluidine blue and immunohistochemical staining of all the groups following proper passive motion are shown in Table II.

Statistically significant differences were observed in the levels of proteoglycans and type II collagen fibers in the cartilage among all the groups following proper passive motion for the early- and middle-stage OA rats (P<0.05). Thus, proper passive motion significantly increased the content of proteoglycans and type II collagen fibers, and the effect was enhanced in cases of prolonged exercise for six weeks. In addition, statistically significant differences were identified in the proteoglycan and type II collagen fiber content between the early- and middle-stage OA experimental groups (P<0.05). Thus, proper passive motion exhibits better repairing effects on articular cartilage in early-stage OA compared with middle-stage OA at the same strength and duration.

### Chondrocyte apoptosis TUNEL staining

Absorbance values from the TUNEL staining apoptosis assays of all the experimental groups following proper passive motion are shown in Table III. Statistically significant differences were identified in the chondrocyte apoptotic rates among all the groups following passive motion in the early-stage OA rats (P<0.05). This result indicated that passive motion significantly reduced the chondrocyte apoptotic rate, and the apoptotic rate was reduced significantly within six weeks. For the middle-stage OA rats, the chondrocyte apoptotic rates in the experimental groups were lower compared with the respective control groups following passive motion, however, no statistically significant differences were observed (P>0.05). In addition, no marked difference was observed between the two experimental groups. These results indicated that six weeks of proper passive motion did not significantly increase the effect of reducing apoptosis with prolonged exercise for the middle-stage OA rats. Statistically significant differences were observed between the early- and middle-stage OA experimental groups (P<0.05), which indicated that proper passive motion had better repairing effects on chondrocytes in early-stage OA compared with middle-stage OA at the same strength and duration.

### Ultrastructure of the articular cartilage

**Figure 1:**
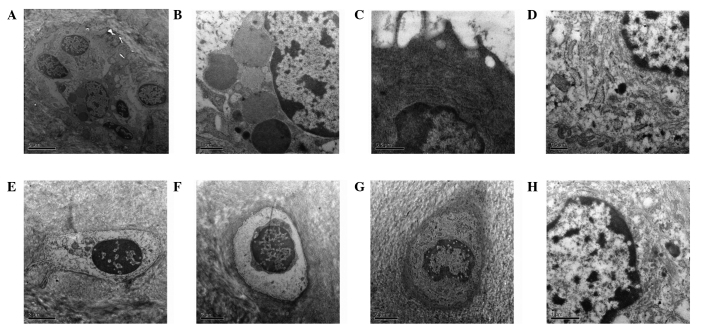
TEM images of the articular cartilage ultrastructure following passive motion in the (A) three- (magnification, ×5,000) and (B) six-week early-stage OA control (magnification, ×12,000), (C) three- and (D) six-week early-stage OA experimental (magnification for both, ×20,000), (E) three- and (F) six-week middle-stage OA control and (G) three- (magnification for all three, ×8,000) and (H) six-week middle-stage OA experimental groups (magnification, × 12,000). TEM, transmission electron microscopy; OA, osteoarthritis.

TEM observations of the articular cartilage ultrastructure following passive motion for all the experimental groups are shown in Fig. 1. In the three-week early-stage OA control group, the cartilage surface exhibited a wave-like, low, flat and rough surface. The number of cytoplasmic organelles was reduced, while the number of thread-like or finely granular matters increased. Projections on the surface of the cells were reduced and the content of collagen fibers decreased. In the six-week early-stage OA control group, partial fracture of the cartilage surface and degeneration of the chondrocytes were observed. In addition, the size of the cells had decreased and cell nuclei were deformed, with chromatin distribution uneven and visible fat droplets. In the three-week early-stage OA experimental group, no apparent nude chondrocytes and collagen were observed. The number of cellular projections increased on the chondrocyte surface, as well as the number of cytoplasmic organelles, with the matrix fibers arranged more tightly. In the six-week early-stage OA exercise group, the chondrocyte surfaces were basically complete and the chondrocyte volume had increased. Projections in the endoplasmic reticulum and on the surface of the cells increased, with a higher frequency of thicker collagen fibers. In the three-week middle-stage OA control group, the collagen texture was indistinct and the connections were blocked. A large gap existed between the chondrocytes and lacuna, and the number of projections on the cell surface had decreased. In the six-week middle-stage OA control group, partial fractures existed on the chondrocyte surfaces and vague collagen texture was observed. The cell outline was unclear or missing, and cell nuclei were deformed or not present. In the three-week middle-stage OA experimental group, the cartilage surface exhibited wave-like, low and flat characteristics, and basically clear collagen texture with an even distribution and connections. Chondrocytes were smaller, showing a small number of mitochondria, endoplasmic reticulum and Golgi bodies. In the six-week middle-stage OA experimental group, fewer cells were observed. A number of the chondrocyte outlines were not distinct and exhibited cracks, and the number of projections on the cell surface were reduced. Unclear collagen texture was enhanced, and the number of collagen fibers was reduced.

### Stretching resistance of the medial collateral ligament of the knee joint combination

Stretching resistances of the knee femur-medial collateral ligament-tibia combination in the rats in all the groups following passive motion are shown in Table IV.

No statistically significant difference was identified in the animal weight between the experimental and control groups. Therefore, the difference in ligament tension caused by weight difference was discounted.

In the early- and middle-stage OA models, the ligament tension in the three-week experimental groups increased significantly compared with the respective control groups (P<0.05). In addition, the ligament tension of the six-week experimental groups increased significantly compared with the respective control and three-week groups (P<0.05). These observations indicated that proper passive motion significantly increased the medial collateral ligament tension in the knee joint and enhanced the stability of the knee joint of rats with OA caused by knee fracture. In addition, the effect was improved with prolonged exercise duration.

## Discussion

The repair of articular cartilage in OA is an issue of great concern to clinicians, since no conclusive treatment for OA has been established to date.

In recent years, motion therapy has been applied extensively in the treatment of OA. Motion therapy is a technique of relieving symptoms of patients or improving their function using mechanical factors. This method is considered as an important nonpharmacological therapy that can improve the repairing effect of degenerated articular cartilage and promote the restoration of articular cartilage morphology. Application of motion therapy can improve muscle strength, increase the activity of the knee joint and improve the motion dysfunction of OA patients (7). OA is a disease that primarily involves the articular cartilage, thus, exercise is required to enhance the metabolism of articular cartilage in order to repair the damaged surface of the cartilage and remove the inflammatory substances (8). The repairing effect of exercise on degenerated articular cartilage has been accepted by a number of scholars. However, the possible effect mechanism of motion therapy on articular cartilage is not well-defined.

In the present study, knee joint fracture was applied to prepare an OA degeneration model. A PT98 rat running device was used to investigate the possible mechanism and effect of proper passive motion on the articular cartilage of rats with various stages of OA. The results indicated that for early-stage OA, following three weeks of exercise, the Mankin rating of the articular cartilage decreased gradually and the thickness of the cartilage layer increased when compared with the control group. In addition, the strength resistance of the medial collateral ligament increased progressively, and the proteoglycan content of the cartilage matrix, type II collagen fibers and cell apoptotic rate evidently decreased. TEM images revealed that the number of projections on the surface of the chondrocytes increased, with basically complete surfaces of cartilage and without nude chondrocytes or collagen. Following six weeks of exercise, the thickness of the cartilage layer and ligament tension, as well as the levels of proteoglycans and type II collagen fibers, increased when compared with the control and three-week experimental groups. Additionally, the apoptotic rate decreased and the Mankin rating was lower when compared with the control group. TEM observations also revealed that the cartilage surface was repaired, since the chondrocytes grew in number and size with markedly increased projections on the cell surface, and matrix fibers were arranged firmly with the number of collagen fibers increasing and becoming thicker. The results demonstrated that proper running can repair the surface of cartilage damaged by early-stage OA, and the degenerated cartilage can be healed and regenerated through exercise. Joint exercise may reduce the occurrence of OA by promoting the regeneration and repair of chondrocytes, which may be a key reason that the less severe the OA disease, the more complete the repair. Salter *et al* (9) demonstrated that joint movement prevented the complications caused by joint fracture and stimulated a full layer of cartilage to be healed. Callus is similar to hyaline cartilage in morphology. A previous study demonstrated that joint movement can generate periodical pressure change in the joint, favorable to the in-joint exchange of nutrients and liquid via the synovial hole, which stimulates chondrocyte metabolism and promotes the synthesis of cartilage matrix protein and internal tissue reconstruction (10).

For the middle-stage OA rats, the cartilage layer following three weeks of exercise became thicker than that of the control group, with increased levels of proteoglycans and type II collagen fibers. In addition, the Mankin rating was lower compared with the control group, but the apoptotic rate was unchanged at this stage. The cartilage layer following six weeks of exercise became thicker than that of the control group, with a significant increase in the levels of proteoglycans and type II collagen fibers. The Mankin rating was also lower compared with the control and three-week groups, although no statistically significant differences were observed with regard to proteoglycan content in the cartilage matrix, type II collagen fiber staining or Mankin rating. The six-week experimental group exhibited increased ligament tension when compared with the control and three-week experimental group, but no significant reduction in the apoptotic rate was observed. TEM observations revealed that the chondrocytes remained smaller, with fewer round projections on the surface. In addition, the cartilage surface was wave-like, low and flat, with partial rough fractures and collagen fiber connections remaining slightly disoriented. Therefore, proper running can improve cartilage matrix damaged by middle-stage OA, although no marked repair in the degenerated chondrocytes was observed. The possible reason is dynamic mechanical loads on articular cartilage are exerted against the effects of the inflammatory mediator, bacterial lipopolysaccharide, and improve the expression of type II collagen and proteoglycans (11). This phenomenon aids the recovery of collagen and proteoglycan, as well as improves the quality of articular cartilage rehabilitation.

The rehabilitation effect of motion on degenerated cartilage is more significant in a knee fracture model, which may be associated with the pathogenesis (12). Articular cartilage degeneration in a knee fracture model is a result of poor nutrition. Proper joint motion can promote the secretion of synovial fluid, which is required to maintain the normal metabolism of articular cartilage. Passive and constant motion therapy is based on periodical press joint (13). This therapy can improve cartilage nutrition, enhance the strength, thickness and elasticity of articular cartilage and inhibit surface rupture and inflammation caused by cartilage denaturation or degeneration. These conditions enhance and recover the maximum load on the extension position of the knee and the stability of the knee joint, as well as significantly relieve pain and benefit load. They can also further increase and strengthen the knee stability and motion function.

OA patients usually suffer from decreased muscle strength around the joint, causing joint instability, which is a risk factor for OA progression (14). Proper motion can increase the muscle strength around the joint, enhance joint instability and relieve pain. The present study demonstrated that the stretching resistances of the medial ligament in the early and middle-stage OA experimental rat models were markedly increased following passive motion. In addition, the ligament tension increased with prolonged exercise duration, which is favorable for the stability of the joint. Deyle *et al* (15) conducted a random control experiment with 83 knee OA patients. The treatment group received four months exercise using a stationary bike, and the results showed that the exercise markedly improved the knee function rating, the degree of pain and stiffness, body activity and the travel distance of the patients. A number of similar studies have been conducted and consistent results have been obtained (16–19). Furthermore, motion can promote systemic and articular local blood circulation, as well as maintain better functional status of the body movement, to further prevent secondary damage to the joint (20).

Individuals with abnormal joint anatomical structure, severe joint damage or past injury, poor joint stability, joint or muscle innervation disorder, poor muscle strength or who are overweight are more prone to suffering from OA. These individuals may benefit from regular physical activity that protects joints from damage, thereby maintaining or increasing muscle strength, harmonization and the overall state of the joint (21).

Thus, the results of the present study demonstrate that proper passive motion can improve the repairing effect of degenerated cartilage, promote the morphological rehabilitation of articular cartilage, improve the metabolism of chondrocytes and delay the degeneration progress of degenerated cartilage. The repairing effect of motion on degenerated cartilage is more significant if the disease is not severe, since joint motion may reduce the occurrence of OA via the promotion of the rehabilitation and repairing of early-stage OA. Proper passive motion has no significant repairing effect on the degenerated chondrocytes for middle- and late-stage OA, since the main effect is to improve the damaged cartilage matrix and enhance the stability of the joint. Thus, proper passive motion can effectively block OA through providing better rehabilitation on the damaged cartilage.

## Figures and Tables

**Table I:** Comparison of cartilage thicknesses and Mankin ratings among the groups (mean ± SD).

	Early-stage OA				Middle-stage OA			
		
	Cartilage thickness (μm)		Mankin rating		Cartilage thickness (μm)		Mankin rating	
				
Subgroup	Control group	Exercise group	Control group	Exercise group	Control group	Exercise group	Control group	Exercise group
3-week	223±23	244±20	4.39±0.97	3.93±1.16	217±25	233±19	8.93±1.73	8.39±1.63
6-week	224±20	271±28	6.35±1.57	4.02±1.19	211±19	249±21	10.75±3.12	10.02±2.57

OA, osteoarthritis.

**Table II:** Comparison of toluidine blue absorbance and immunohistochemical staining following passive motion (mean ± SD).

	Early-stage OA				Middle-stage OA			
		
	Proteoglycans		Type II collagen fibers		Proteoglycans		Type II collagen fibers	
				
Subgroup	Control group	Exercise group	Control group	Exercise group	Control group	Exercise group	Control group	Exercise group
3-week	0.33±0.06	0.48±0.05	0.36±0.05	0.42±0.08	0.29±0.04	0.35±0.09	0.32±0.03	0.37±0.09
6-week	0.31±0.05	0.55±0.04	0.35±0.05	0.54±0.09	0.28±0.05	0.39±0.06	0.38±0.02	0.43±0.05

OA, osteoarthritis.

**Table III:** Comparison of TUNEL staining absorbances of the articular cartilages following passive motion (mean ± SD).

	Early-stage OA		Middle-stage OA	
		
Group	3-week	6-week	3-week	6-week
Control	0.41±0.03	0.47±0.05	0.53±0.04	0.58±0.07
Experimental	0.26±0.02	0.22±0.02	0.49±0.07	0.55±0.03

OA, osteoarthritis; TUNEL, terminal deoxynucleotidyl transferase dUTP nick end labeling.

**Table IV:** Comparison of medial collateral ligament combination stretching resistances following passive motion (mean ± SD).

	Early-stage OA				Middle-stage OA			
		
	Stretching force (N)		Weight (g)		Stretching force (N)		Weight (g)	
				
Subgroup	Control group	Exercise group	Control group	Exercise group	Control group	Exercise group	Control group	Exercise group
3-week	14.91±1.45	18.62±1.19	225±15	221±16	14.25±1.07	16.51±1.22	279±25	278±18
6-week	15.20±1.61	20.25±1.22	261±16	266±22	13.92±1.16	18.37±1.16	310±35	313±37

OA, osteoarthritis.

## References

[1] Wang YB, Wang HF (2012). Rehabilitation-solution of full treatment of knee joint osteoarthritis. Chin J Rehabil Med.

[2] Gu Y, Dai K, Qiu S (1995). A morphological study of degenerative mechanism of articular cartilage by abnormal high stress. Chinese Journal of Orthopaedics.

[3] Moran ME, Kim HK, Salter RB (1992). Biological resurfacing of full-thickness defects in patellar articular cartilage of the rabbit. Investigation of autogenous periosteal grafts subjected to continuous passive motion. J Bone Joint Surg Br.

[4] Brismée JM, Paige RL, Chyu MC (2007). Group and home-based tai chi in elderly subjects with knee osteoarthritis: a randomized controlled trial. Clin Rehabil.

[5] Zhang H, Jiang HP, Wang DP (1848). Discussion of osteoarthritic animal model get from immobilized knees of rabbit in full extension using plaster cast. China Journal of Modern Medicine.

[6] Mankin HJ, Dorfman H, Lippiello L, Zarins A (1971). Biochemical and metabolic abnormalities in articular cartilage from osteo-arthritic human hips. II Correlation of morphology with biochemical and metabolic data. J Bone Joint Surg.

[7] Maurer BT, Stern AG, Kinossian B, Cook KD, Schumacher HR (1999). Osteoarthritis of the knee: isokinetic quadriceps exercise versus an educational intervention. Arch Phys Med Rehabil.

[8] Huang MH, Lin YS, Yang RC, Lee CL (2003). A comparison of various therapeutic exercises on the functional status of patients with knee osteoarthritis. Semin Arthritis Rheum.

[9] Salter RB, Simmonds DF, Malcolm BW, Rumble EJ, MacMichael D, Clements ND (1980). The biological effect of continuous passive motion on the healing of full-thickness defects in articular cartilage. An experimental investigation in the rabbit. J Bone Joint Surg Am.

[10] Lee MS, Ikenoue T, Trindade MC (2003). Protective effects of intermittent hydrostatic pressure on osteoarthritic chondrocytes activated by bacterial endotoxin in vitro. J Orthop Res.

[11] Shimizu T, Videman T, Shimazaki K, Mooney V (1987). Experimental study on the repair of full thickness articular cartilage defects: effects of varying periods of continuous passive motion, cage activity, and immobilization. J Orthop Res.

[12] Sandoval R (2011). Proximal femur fracture in a patient referred to a physical therapist for knee pain. J Orthop Sports Phys Ther.

[13] Bonutti P, Marulanda GA, McGrath MS (2010). Static progressive stretch improves range of motion in arthrofibrosis following total knee arthroplasty. Knee Surg Sports Traumatol Arthrosc.

[14] Mangani I, Cesari M, Kritchevsky SB (2006). Physical exercise and comorbidity. Results from the Fitness and Arthritis in Seniors Trial (FAST). Aging Clin Exp Res.

[15] Deyle GD, Henderson NE, Matekel RL, Ryder MG, Garber MB, Allison SC (2000). Effectiveness of manual physical therapy and exercise in osteoarthritis of the knee. A randomized, controlled trial. Ann Intern Med.

[16] Lin SY, Davey RC, Cochrane T (2004). Community rehabilitation for older adults with osteoarthritis of the lower limb: a controlled clinical trial. Clin Rehabil.

[17] Foley A, Halbert J, Hewitt T, Crotty M (2003). Does hydrotherapy improve strength and physical function in patients with osteoarthritis - a randomised controlled trial comparing a gym based and a hydrotherapy based strengthening programme. Ann Rheum Dis.

[18] Xuan Y, Lu YL, Li J (2003). Exercise therapy in osteoarthritis of the knee. Chinese Journal of Rehabilitation Medicine.

[19] Liu WM (2003). The effect of isokinetic exercise on function and symptoms of knee joint osteoarthritis patients. Chinese Journal of Clinical Rehabilitation.

[20] Pincivero DM, Lephart SM, Karunakara RG (1997). Relation between open and closed kinematic chain assessment of knee strength and functional performance. Clin J Sport Med.

[21] Buckwalter JA, Mankin HJ (1998). Articular cartilage: degeneration and osteoarthritis, repair, regeneration, and transplantation. Instr Course Lect.

